# John Hunter

**Published:** 1898-03

**Authors:** 


**John Hunter.** By STEPHEN PAGET. With Introduction by Sir JAMES PAGET. Pp. 272. London: T. Fisher Unwin. 1897.

Our first word in reference to this book must be one of sincere regret at the death of Mr. Ernest Hart, the accomplished editor of the series of biographies of which this volume is the introductory one. No more able journalist or energetic and wise reformer has ever been found in the world of medicine, and his removal leaves a space in our ranks, which it will be difficult to adequately fill.

No name more appropriate wherewith to begin this series of "Masters of Medicine" could have been found than that of John Hunter; and no one could be found so suitable to write the short introductory chapter as the acknowledged and venerable head of the surgical profession in this country. Sir James Paget refers, in terms most suggestive of a definite fellow-feeling, to Hunter's natural love of collecting and observing, and his belief that truth in science is to be attained not so much by thinking out deductions from facts hitherto admitted, as by continued experiments and the collection of more material and more facts, or, in the words of Hunter himself, "Don't think, try;" and although year after year some chosen man of high standing in our profession has searched out all possible records of John Hunter, in the hope of finding some new topic wherewith to garnish his Oration in Lincoln's Inn Fields—and he has been studied from every point of view—there was yet room for this pleasant little book.

John Hunter was born in Scotland on the 13th of February, 1728, the youngest son of a large and talented family. When he was thirteen years old he lost his father, and he became the spoilt child of his mother. He is a very remarkable, or almost unique, instance where an idle or illiterate boy, who would not learn but spent his time in wandering about apparently without aim and unfit for a useful life—for he was tried on more than one occasion,—became a most hardworking and renowned naturalist, philosopher, and surgeon, leaving volumes of valuable research behind him, with the credit, as Sir James Paget says, "of having raised our profession from the condition in which he found it, a mechanical art, to one grounded on scientific principles."

We have in these pages a pleasing account of the Hunter family, who were evidently above the average in mental capacity; and it is satisfactory to find justice done to the eldest brother, William, who gave John, many years his junior, his first chance of success. In 1748 John went to London and joined his brother William, who had been settled there for many years. The brothers became involved in many controversies, and, in after days, there was complete estrangement between them.

John soon recognised that surgery, for which he had a great taste, was to be his profession in life. His brother had got him permission to attend the hospital at Chelsea, where Cheselden was then the chief; and he attended from 1749 to 1751, doing a little minor surgery. At the latter date Cheselden died, and he then entered St. Bartholomew's under Percival Pott; and, finally, in 1754, with the view of getting ultimately on its staff, he entered at St. George's, and was sole house-surgeon in 1756.

In 1760 his health broke down, and he was threatened with consumption; and obtaining a commission as army surgeon he went abroad, and was employed both in naval and military matters. He saw service in Belle Isle and Spain and Portugal, and after an absence of about two years returned in good health to London to resume his work.

Hunter's numerous preparations and the living animals which he kept increased to such an extent that he was obliged to look out for a home for them, which he found at Earl's Court, a hamlet "about two miles from London." There is an interesting account of his obtaining the body of the Irish giant, Byrne, after setting men to watch for his death, and bribing the men who watched, to whom he had to pay £500; and transferring it in his own carriage through the London streets at night. Some have confused this giant with our local giant, O'Brien, who was 8 feet 3 inches in height (a description of whom the writer has procured from one who had seen him). He died in 1805, and, although attempts were made to get the body, was safely buried in Trenchard Street Roman Catholic Church.

In 1768 John was elected surgeon to St. George's; and in 1771 he married. The hospital appointment gave him an opportunity of taking house-pupils, of whom he had a succession; and his popularity with the students, and the numerous fees resulting from it, led to much jealousy with his colleagues. One of his early pupils was Edward Jenner, and between the two a continued close friendship existed.

Hunter's first course of lectures on the "Principles of Surgery" was given in 1773. In 1785 he performed successfully his first operation for popliteal aneurysm, by ligature of the femoral at a distance from the disease: and it is an instance of the petty jealousies of his colleagues in some of their frequent encounters with Hunter that they put forward this case as an instance of his incapacity, as the patient had died; but they omitted to say that he died a year after of an entirely different complaint.

Hunter had suffered for many years from angina, and from an attack of this he died on the 16th of October, 1793, at a meeting of the governors of St. George's Hospital.

We have a description of Hunter's appearance: "He was five feet two inches high, strongly built, and toward the end of his life somewhat corpulent"; a description which does not seem to tally well with the celebrated picture by Reynolds, which represents him as certainly a slight if not tall man. His character is touched upon: his hasty temper, his bluntness and his caustic humour, as well as his generosity and pleasant style of conversation, and a few incidents are given to illustrate these several features. He is contrasted with Ambroise Paré. "Paré advanced the art of surgery, but Hunter taught the science of it."

The purchase of the museum by the Government, and its transference to the College in Lincoln's Inn Fields, together with other details, and the destruction of his manuscripts by his executor, Sir Everard Home, occupy the final chapter of a pleasant and well-written book; and we shall welcome others of the series.

